# Artificial intelligence-based CT histogram parameters differentiating bronchiolar adenoma and lung adenocarcinomas: A two-center study

**DOI:** 10.1371/journal.pone.0331336

**Published:** 2025-09-08

**Authors:** Wen Zhao, Ziqian Zhao, Yingxia Wang, Haiyan Yang, Weiyuan Zhang, Jianyou Chen, Xinhui Yang, Zhijie Duan, Fengyi Li, Zhiquan Han, Xin Zhang, Zhilin Li, Dan Han, Tengfei Ke

**Affiliations:** 1 Department of Medical imaging, the First Affiliated Hospital of Kunming Medical University, Yunnan, Kunming, China; 2 Department of Pathology, the First Affiliated Hospital of Kunming Medical University, Yunnan, Kunming, China; 3 Department of Ultrasound, Chongqing General Hospital, Chongqing, China; 4 Department of Radiology, the Third Affiliated Hospital of Kunming Medical University, Yunnan, Kunming, China; 5 GE Healthcare, Shanghai, China; Tokyo Medical University: Tokyo Ika Daigaku, JAPAN

## Abstract

**Purpose:**

Bronchiolar adenoma (BA) is a rare benign pulmonary neoplasm originating from the bronchial mucosal epithelium and mimics lung adenocarcinoma (LAC) both radiographically and microscopically. This study aimed to develop a nomogram for distinguishing BA from LAC by integrating clinical characteristics and artificial intelligence (AI)-derived histogram parameters across two medical centers.

**Methods:**

This retrospective study included 215 patients with diagnoses confirmed by postoperative pathology from two medical centers. Medical center 1 provided 151 patients (68 BA and 83 LAC nodules) as the training cohort, while medical center 2 contributed 64 patients (28 BA and 36 LAC nodules) as the external validation cohort. Risk predictors and the nomogram were developed using clinical characteristics and AI-derived histogram parameters.

**Results:**

Nodule density (solid, ground glass, and subsolid) exhibited a statistically significant difference between the BA and LAC groups (p < 0.01). The following parameters were significantly higher in the LAC group compared to the BA group (all p < 0.05): 2D long diameter, 2D short diameter, 2D average diameter, 2D maximum surface area, 3D long diameter, 3D surface area, 3D volume, and entropy. In contrast, CT value variance was significantly lower in the LAC group than in the BA group (p < 0.01). A nomogram was constructed incorporating density, 2D short diameter, and CT value variance. The area under the curve (AUC) of the nomogram in the training and validation cohorts were 0.821, 0.811.

**Conclusion:**

The AI-based nomogram, as a non-invasive preoperative tool, had the potential to enhance diagnostic accuracy for distinguishing BA from LAC.

## Introduction

The widespread use of computed tomography (CT) and high-resolution CT (HRCT) in chest imaging has significantly enhanced the detection of solitary pulmonary nodules (SPNs) [1]. CT has become a cornerstone in the early detection of lung cancer [2]. Concurrently, the detection of benign pulmonary nodules has also increased. However, a major challenge in clinical practice remains the accurate differentiation of benign pulmonary nodules from lung adenocarcinoma (LAC)because their CT features are often similar.

Bronchiolar adenoma (BA), a benign pulmonary tumor originating from the bronchial mucosal epithelium, mimics LAC in both CT imaging and intraoperative frozen sections [3,4]. The 5th edition of the World Health Organization (WHO) classification of lung tumors (2021) recognized BA/ciliated mucinous papillary tumor (CMPT) as a distinct type of benign tumor [5]. In 2018, Chang et al. introduced the concept of BA, suggesting that CMPT is a subtype of BA [6]. The primary histological feature that distinguishes BA from LAC is the presence of a characteristic double-layered cellular structure in BA, which is absent in LAC [7]. However, detecting this double-layered architecture requires immunohistochemical (IHC) staining for markers such as p40 and CK5/6, making intraoperative frozen section diagnosis challenging [8]. Therefore, enhancing the accuracy of preoperative BA diagnosis is especially important to prevent unnecessary escalation of wedge resections to segmental resections or lobectomies.

In recent years, artificial intelligence (AI) has made significant strides as a noninvasive tool for the screening and diagnosis of lung nodules [9,10]. Voxel-based CT histogram analysis, a core technique in radiomics, provides critical radiological features that assess tumor heterogeneity beyond the capabilities of visual inspection by clinicians [11,12]. AI-assisted CT screening algorithm could automatically localize and identify lung nodules, while extracting corresponding voxel-based histogram parameters, alleviating the heavy workload of radiologists. Some studies have shown the clinical value of voxel-based histogram analysis in preoperative diagnosis, assessing invasiveness, predicting lymph node involvement, and evaluating treatment responses in lung cancer patients [13–16]. Due to the relatively recent recognition of BA and the limited research available, especially in the fields of AI and radiomics, the application of these technologies to BA remains largely unexplored. A recent study showed that CT texture analysis holds promise in distinguishing BA from LAC included only 15 patients with BA from a single center a single-institution [17]. In contrast, our study enrolled 215 patients (96 with BA and 119 with LAC) from two independent medical centers, with the second center serving as an independent external validation cohort.

In this study, we aimed to leverage AI platform based on voxel histogram algorithms to identify key features and construct a nomogram for distinguishing between BA and LAC across two medical institutions.

## Methods

### Patients

This retrospective study was approved by the ethics committee of the 2 participating hospitals (NO.2024-L-169), and the informed consent requirement of patients was waived.We retrospectively collected patients who presented with pulmonary nodules on CT images and were subsequently diagnosed with BA or LAC through surgical pathology biopsy between 01/01/2021 and 31/12/2024 at medical center 1 and medical center 2, we completed the collection of these cases from 1/2/2025–28/2/2025 and included them in the study. The inclusion criteria were as follows: (1) availability of complete clinical and medical data, (2) confirmation of BA or LAC by pathology following surgical resection, (3) preoperative CT was performed < 2 weeks before surgery, (4) pulmonary nodule diameter ≤ 3 cm on CT imaging, (5) CT slice thickness ≤ 1.5 mm. The exclusion criteria were: (1) inconclusive pathological diagnosis based on needle biopsy or bronchoscopy, (2) preoperative CT artifacts are significant, (3) malignant tumors with other organs, (4) a history of chest surgery.

**Fig 1:**
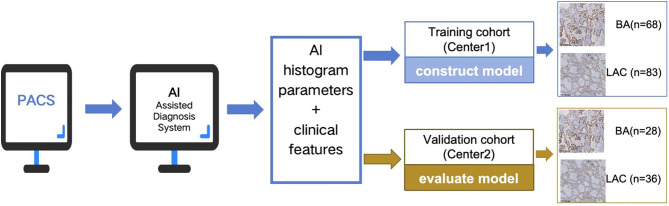
The overview of this study methodology.

In this study, one nodule was selected for each patient, with the largest nodule chosen for patients presenting with multiple nodules. A total of 151 patients from medical center 1 were included in the training cohort, comprising 68 BA and 83 LAC nodules. The independent external validation cohort consisted of 64 patients from medical center 2, including 28 BA and 36 LAC nodules. To match the number of BA cases, the LAC group was adjusted through random sampling using Python (sample size ratio between LAC and BA ranged from 1.2 to 1.3). An overview of the study methodology was presented in Fig 1.

### CT examinations

All patients underwent the chest CT scan with revolution CT (GE Healthcare), IQON CT 256 unit (Philips Healthcare) and dual-energy CT (Siemens Healthcare). The imaging acquisition parameters were as follow: 120 kV tube voltage, 50–250 mA effective tube current or tube current modulation, 0.5–1.0s rotation time, 1.0–1.5 pitch, conventional thin-layer thickness of 5.0 mm, and reconstruction layer thickness of 1.0 mm or 1.5 mm. The CT images were reviewed with standard lung window settings (window width 1600HU, window level −600HU) and mediastinal window settings (window width 400HU, window level 40HU).

### Clinical characteristics and AI-derived parameters

The clinical characteristics of the participants were obtained from the hospital information system (HIS), including gender, age, family history, and smoking history. All conventional thin-layer images in digital imaging and communications in medicine (DICOM) format were transmitted to the lung nodule AI assisted diagnosis system (Version 2.4.0, Deepwise Technology, Beijing, China) for automatic detection and labeling of all lung nodules. The lung nodule AI platform is based on a recurrent convolutional neural network (CNN) algorithm, enabling automatic segmentation of nodules. The system automatically delineates the boundaries of the nodules and calculates the number of voxels corresponding to each CT value within the entire nodule. Each CT value and its corresponding voxel count are stored as a list, and the complete set of lists for the entire nodule is stored as a dictionary. The required parameters are then derived from this dictionary using corresponding formulas. Firstly, the CT value threshold of −300HU was used to differentiate density type, including solid, pure ground-glass, and part-solid densities. Subsequently, histogram parameters were generated by the AI software based on voxel intensity histogram features were automatically calculated using Python and the corresponding formulas, as follows: (1) two-dimensional (2D) long diameter, (2) 2D short diameter, (3) 2D average diameter [(long diameter + short diameter)/ 2], (4) 2D maximum surface area, (5) three-dimensional (3D) long diameter, (6) 3D surface area, (7) 3D volume, (8) maximum CT value, (9) minimum CT value, (10) mean CT value, (11) CT value variance, (12) sphericity, (13) kurtosis, (14) skewness, (15) energy, (16) compactness, and (17) entropy. All 2D measurements were taken from the largest cross-section, while the 3D measurements were derived from the largest elliptical sphere with the tumor’s maximum diameter. Sphericity and compactness (ranging from 0 to 1) reflect nodule morphology, with lower values indicating more irregular and potentially more invasive lesions. Kurtosis and skewness reflect the uniformity of nodule density by characterizing the fluctuation range and asymmetry of CT values, respectively. Energy measures the magnitude of voxel values in the image. Entropy measures the complexity of image texture, increasing with higher pathological grade and greater infiltration, which make the nodule texture more complex.

### Statistical analysis

The data were statistically analyzed using SPSS (version 26.0), Python (version 3.7.12), and R software (version 4.1.0). Categorical data were expressed as frequencies (percentages), and the chi-square test was used for comparisons between the two groups. For continuous data, were assessed using the t-test for normally distributed data, with results presented as mean ± standard deviation. For non-normally distributed data, the Mann–Whitney U test was applied, with results presented as median (interquartile range, IQR). In the training cohort, univariable logistic regression was used to screen relevant features. The relevant features were identified using backward stepwise multivariable logistic regression. A predictive model and nomogram were constructed based on these final relevant features. The 5-fold cross-validation method was used to evaluate the performance of the predictive model in the training data. Receiver operating characteristic (ROC) curves and the area under the curve (AUC) were calculated to assess the model’s accuracy. In the external validation cohort, the ROC and AUC were also measured to evaluate the performance of the model. The calibration curve and decision curve analysis (DCA) were obtained. The p-value < 0.05 was considered statistically significant.

## Results

### Clinical characteristics

A total of 215 patients were enrolled in this study (Table 1), including 74 males and 141 females. The cohort consisted of 151 patients in the training cohort (medical center 1) and 64 patients in the validation cohort (medical center 2). There were no statistically significant differences between the BA and LAC groups in terms of gender, age, smoking history, and family history (all p > 0.05) in both the training and validation datasets (Table 1).

**Table 1:** Clinial characteristics and AI histogram parameters in the training and validation cohorts.

	Training cohort (n = 151)			Validation cohort (n = 64)		
	BA(n = 68)	LAC(n = 83)	*p*	BA(n = 28)	LAC(n = 36)	*p*
Clinical characteristics						
Gender			0.224			0.771
Male	22 (32.4%)	36 (43.4%)		8 (28.6%)	8 (22.2%)	
Female	46 (67.6%)	47 (56.6%)		20 (71.4%)	28 (77.8%)	
Age, years	56.0 [46.8, 63.5]	56.0 [45.5, 63.5]	0.874	57.5 [51.8, 67.8]	54.0 [41.0, 62.0]	0.057
Family history			0.074			1
Yes	67 (98.5%)	76(91.6%)		25 (89.3%)	32 (88.9%)	
No	1 (1.47%)	7 (8.43%)		3 (10.7%)	4 (11.1%)	
Smoking history			0.381			0.078
Yes	55 (80.9%)	61 (73.5%)		23 (82.1%)	35 (97.2%)	
No	13 (19.1%)	22 (26.5%)		5 (17.9%)	1 (2.78%)	
**Histogram features**						
Density			0.001*			<0.001*
Solid	51 (75.0%)	37 (44.6%)		22 (78.6%)	10 (27.8%)	
Ground glass	9 (13.2%)	24 (28.9%)		3 (10.7%)	13 (36.1%)	
Subsolid	8 (11.8%)	22 (26.5%)		3 (10.7%)	13 (36.1%)	
Location			0.001*			0.194
Left upper lobe	8 (11.8%)	22 (26.5%)		4 (14.3%)	11 (30.6%)	
Left lower lobe	23 (33.8%)	12 (14.5%)		7 (25.0%)	9 (25.0%)	
Right upper lobe	8 (11.8%)	22 (26.5%)		3 (10.7%)	7 (19.4%)	
Right middle lobe	3 (4.41%)	7 (8.43%)		1 (3.57%)	0 (0.00%)	
Right lower lobe	26 (38.2%)	20 (24.1%)		13 (46.4%)	9 (25.0%)	
**Histogram parameters**						
2D long diameter, mm	7.50 [5.00, 11.0]	14.0 [8.00, 17.5]	<0.001*	7.50 [5.00, 11.0]	9.50 [7.00, 16.0]	0.021*
2D short diameter, mm	6.00 [4.00, 8.00]	9.00 [7.00, 13.0]	<0.001*	6.00 [4.00, 8.00]	7.50 [6.00, 11.0]	0.008*
2D average diameter, mm	7.00 [5.00, 10.0]	12.0 [8.00, 15.0]	<0.001*	7.50 [5.00, 9.25]	9.00 [7.00, 14.0]	0.017*
2D maximum surface area, mm^2^	30.3 [15.7, 64.2]	97.6 [40.6, 152]	<0.001*	31.5 [15.6, 56.2]	53.6 [31.1, 125]	0.012*
3D long diameter, mm	8.81 [6.65, 12.1]	14.6 [9.42, 19.0]	<0.001*	8.24 [6.27, 12.5]	11.1 [7.82, 16.7]	0.017*
3D surface area, mm^2^	148 [97.2, 281]	441 [210, 748]	<0.001*	145 [93.9, 255]	260 [144, 612]	0.005*
3D volume, mm^3^	180 [97.5, 450]	888 [267, 1555]	<0.001*	167 [103, 393]	374 [193, 1340]	0.007*
maximum CT value, HU	266 [40.5, 445]	259 [28.5, 398]	0.558	230 [9.25, 529]	222 [18.0, 423]	0.607
minimum CT value, HU	−1001.00 [-1024.00, -954.75]	−1014.00 [-1024.00, -942.50]	0.741	−1020.50 [-1024.00, -960.25]	−1018.50 [-1024.00, -989.25]	0.961
mean CT value, HU	−350.55 [-528.72, -177.15]	−427.10 [-622.15, -148.20]	0.314	−378.10 [-517.68, -190.50]	−508.40 [-615.35, -233.68]	0.343
CT value variance	65120 [40310, 93085]	43044 [26759, 62411]	0.001*	76859 [38217, 100636]	50992 [32359, 63975]	0.012*
Sphericity	1.00 [0.95, 1.00]	0.97 [0.90, 1.00]	0.031*	1.00 [0.97, 1.00]	0.99 [0.93, 1.00]	0.078
Kurtosis	4.00 [2.52, 8.78]	5.21 [3.94, 9.80]	0.005*	3.67 [2.50, 10.7]	4.66 [2.85, 7.06]	0.989
Skewness	−1.10 [-1.90, -0.82]	−1.63 [-2.39, -1.06]	0.019*	−1.10 [-2.13, -0.66]	−1.28 [-1.98, -0.70]	0.973
Energy (×10^10^)	2.10 [3.95, 9.82]	26.01 [5.50, 135.00]	<0.001*	1.30 [0.59, 5.28]	11.55 [1.88, 104.32]	0.001*
Compactness	1.00 [0.88, 1.00]	0.92 [0.74, 1.00]	0.014*	1.00 [0.90, 1.00]	0.98 [0.80, 1.00]	0.055
Entropy	7.84 [7.18, 8.77]	8.90 [8.04, 9.64]	<0.001*	8.02 [7.08, 8.85]	8.64 [7.80, 9.42]	0.023*

BA: bronchiole adenoma group; LAC: lung adenocarcinomas group.

*:*p* value was less than 0.05.

### AI features and histogram parameters

There was a statistically significant difference in the density of nodules between the BA and LAC groups. Pure ground-glass and part-solid densities were more likely to be associated with LAC, while solid density was more observed in BA. The lung lobe location of nodules showed a significant difference in the training cohort, but no statistically significant difference was observed in the validation cohort (Table 1).

The 2D long diameter, 2D short diameter, 2D average diameter, 2D maximum surface area, 3D long diameter, 3D surface area, 3D volume, and entropy were significantly higher in the LAC group compared to the BA group in both the training and validation cohorts. In contrast, CT value variance was significantly higher in the BA group than in the LAC group in both cohorts. Energy was higher in the LAC group than in the BA group in the training cohort, but this relationship was reversed in the validation cohort. Sphericity, kurtosis, skewness, and compactness showed statistically significant differences between the two groups only in the training cohort (Table 1). The clinical relevance of the key distinguishing features between BA and LAC was shown in S1 Table.

### Construction and evaluation of predictive model and nomogram

**Fig 2:**
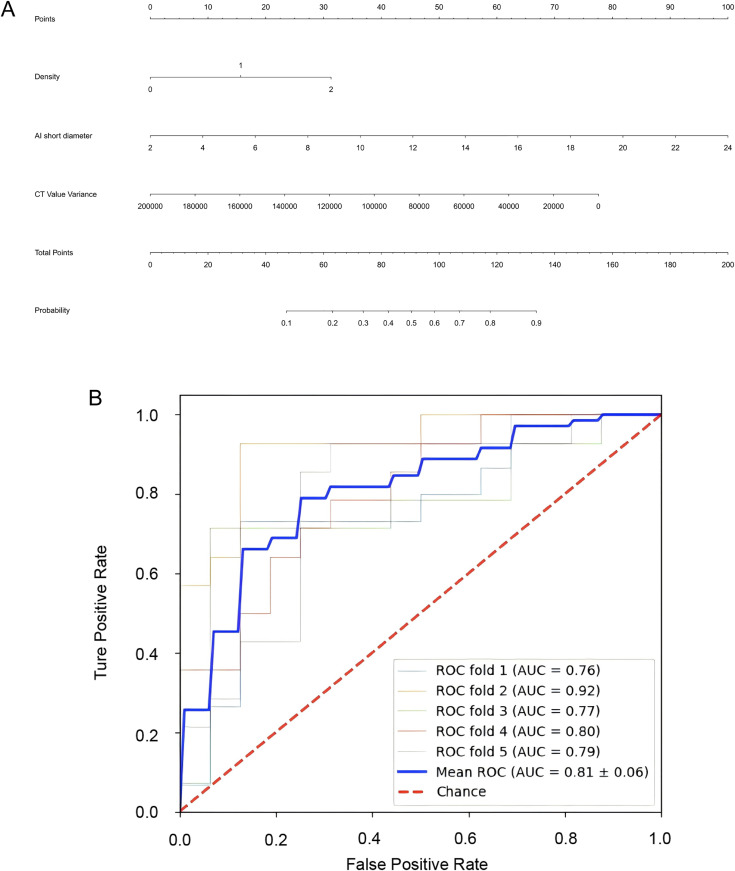
The nomogram for LAC prediction. **(A)** Nomogram developed for the prediction of LAC, which incorporates density, 2D short diameter, CT value variance. **(B)**The ROC curves of the predictive nomogram for distinguishing LAC from BA, using five-fold cross validation in training cohort. *: Density of the nomagram: 0 is solid, 1is ground glass, 2 is subsolid.

The results of the univariate and multivariate logistic regression analyses for risk predictors in the training cohort are presented in Table 2. Variables with a p-value < 0.05 were selected for analysis. In the univariate analysis, 12 factors were identified as risk predictors, including density, 2D long diameter, 2D short diameter, 2D average diameter, 2D surface area, 3D long diameter, 3D surface area, 3D volume, CT value variance, sphericity, compactness, and entropy. High-correlation factors were selected from these variables using backward-stepwise multivariate logistic regression. The results revealed that density, 2D short diameter, and CT value variance were independent predictors for differentiating BA from LAC. Consequently, a model and nomogram were constructed (Fig. 2) using these three variables in the training set, as follow: *Logit (P) = −1.120 + 0.795 Density + 0.231 2D short diameter – 0.00002 CT value variance.*

**Table 2:** Risk predictors in the univariate and multivariate logistic regression analysis.

	OR (95%CI)	*p*	OR (95%CI)	*p*
Density	2.17 (1.38, 3.42)	<0.001	2.22 (1.33, 3.68)	0.001
2D long diameter	1.11 (1.05, 1.18)	<0.001		
2D short diameter	1.20 (1.10, 1.32)	<0.001	1.26 (1.14, 1.39)	< 0.001
2D average diameter	1.15 (1.07,1.24)	<0.001		
2D maximum surface area	1.01(1.00, 1.01)	0.001		
3D long diameter	1.09 (1.04, 1.15)	<0.001		
3D surface area	1.00(1.00, 1.00)	0.005		
3D volume	1.00 (1.00, 1.00)	0.030		
CT value variance	1.00 (1.00, 1.00)	0.001	1.00 (1.00, 1.00)	< 0.001
Sphericity	0.00 (0, 0.68)	0.035		
Compactness	0.07 (0.01, 0.61)	0.016		
Entropy	1.94 (1.44, 2.61)	<0.001		

OR: odds ratio; CI: confidence interval.

**Fig 3:**
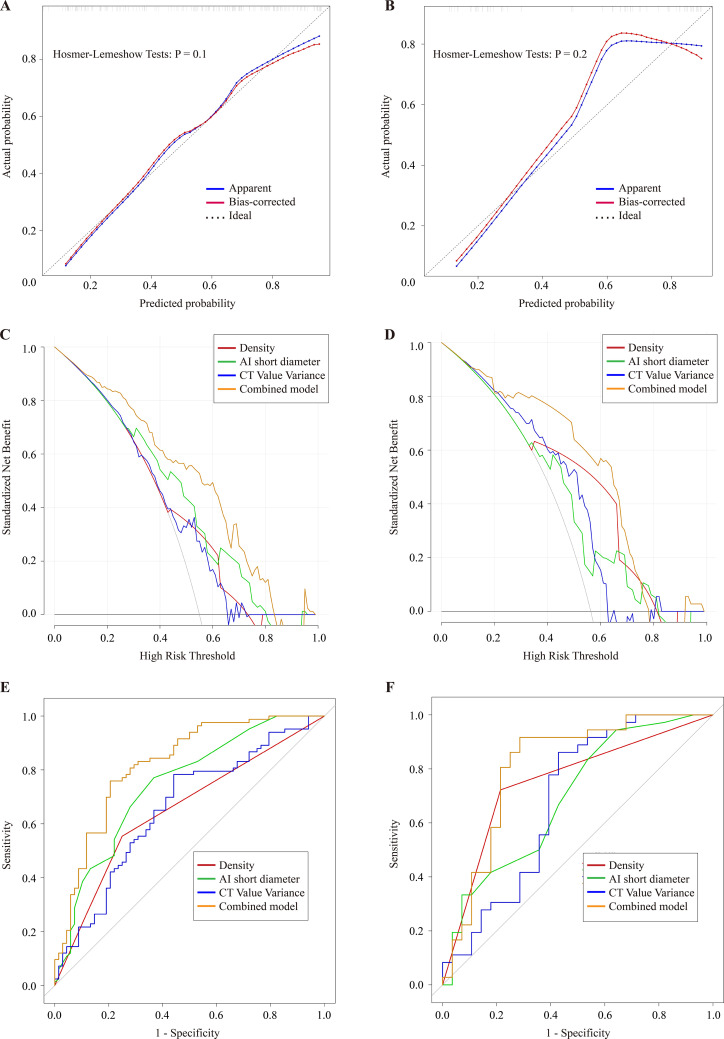
The calibration curve of the nomogram is shown in the training (A) and validation (B) datasets. The DCA curves of the nomogram for the training (C) and validation (D) datasets, the x-axis and y-axis represent the threshold probability and the net benefit, respectively. The ROC curves of the density, 2D short diameter, CT value variance and the nomogram in training (E) and validation (F) datasets.

**Fig 4:**
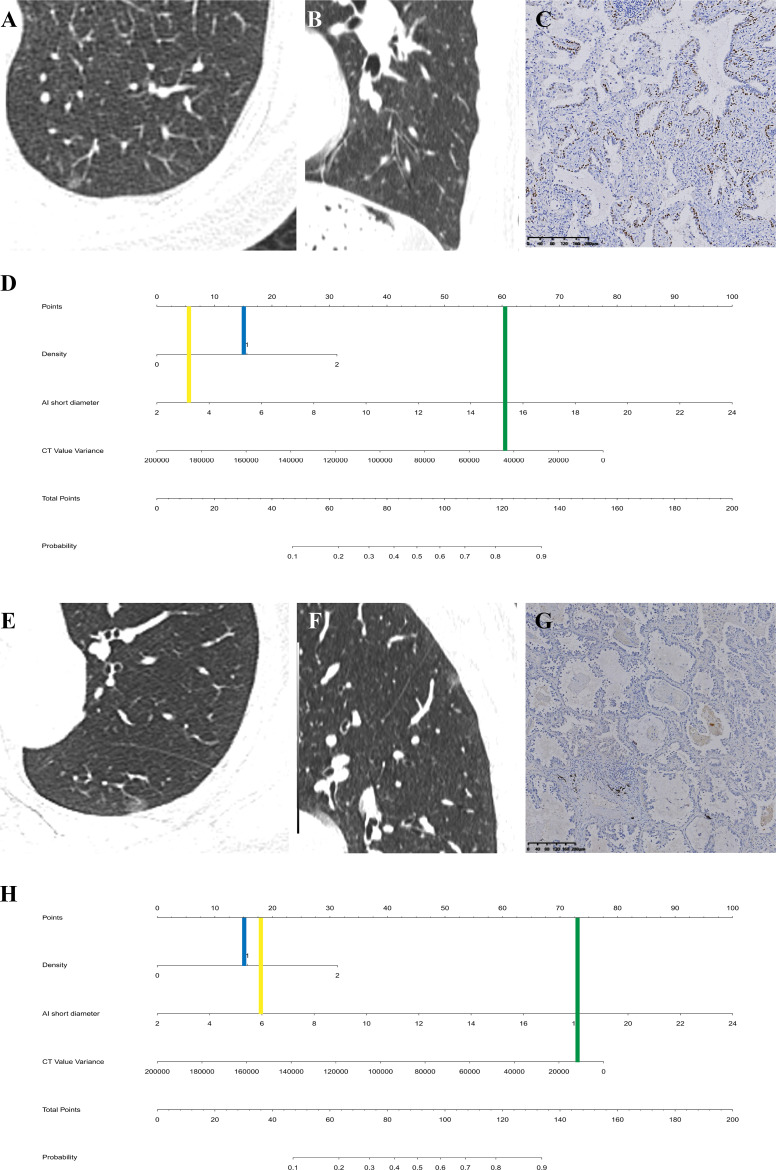
(A-D) Patient 1, BA in a 58-year-old female. CT images (A axial view, B sagittal view) showed a pure ground-glass nodule in the left lung lower lobe. The 2D short diameter and CT value variance of the nodule were 3 mm and 43254.8 by AI, respectively. Immunohistochemistry (×200) showed positive staining of basal cells for p63 oncogene **(C)**. Vertical lines of each variable were drawn in the nomogram **(D)**, and to obtain the total points was 80. The graph revealed that the risk of LAC about 35%. **(E-H)** Patient 2, LAC in a 53-year-old female. CT images (E axial view, F sagittal view) showed a pure ground-glass nodule in the left lung lower lobe, mimics patient 1. The 2D short diameter and CT value variance of the nodule were 6 mm and 11918.8 by AI, respectively. Immunohistochemistry (×200) showed negative staining of basal cells for p63 oncogene **(G)**. Vertical lines of each variable were drawn in the nomogram **(H)**, and to obtain the total points was 106. The graph revealed that the risk of LAC > 70%. *Density: blue line; 2D short diameter: orange line; CT value variance: green line.

The AUC of this nomogram was analyzed using 5-fold cross-validation in the training cohort, resulting in an AUC of 0.81 ± 0.06 (Fig 2). The predictive value of the nomogram was evaluated using calibration curve, ROC curve, and DCA, as shown in Fig 3. The calibration curve indicated good predictive accuracy, with p-values of 0.100 and 0.200 in the training and validation cohorts, respectively (Fig 3). The AUC of the predictive model was 0.821 (95% CI: 0.753–0.890) and 0.811 (95% CI: 0.693–0.928) in the training and validation cohorts, respectively, as shown in Fig 3 and Table 3. The De-Long test indicated that the differences in AUC between the nomogram and the individual predictors (density, 2D short diameter, and CT value variance) were statistically significant (P < 0.05). The sensitivity, specificity, and accuracy of the nomogram in the training and validation datasets were 0.759, 0.794, 0.775 and 0.778, 0.786, 0.781, respectively. Finally, the DCA demonstrated that the nomogram provided greater clinical application value and net benefit at higher risk threshold probabilities (Fig 3). As demonstrated by the DCA, the nomogram exhibited the highest standardized net benefit across a wide range of high-risk thresholds, particularly between 0.1 and 0.8 both in the training and validation cohorts. This suggests that the nomogram can assist clinicians in more accurately assessing patient prognosis. Since the nomogram was constructed based on multiple prognostic factors, it outperformed any single predictor alone. Sample cases of the diagnostic use of the nomogram were shown in Fig 4.

**Table 3:** ROC curve analysis for identifying BA from LAC in the training and validation cohorts.

	AUC (95%CI)	Acc	Spe	Sen	Threshold	PPV	NPV	Precision	DeLong test (vs. model)
Training cohort									
Density	0.653 0.576-0.730)	0.642	0.750	0.554	0.534	0.730	0.580	0.730	* < 0.001
2D short diameter	0.746 (0.667-0.825)	0.709	0.632	0.771	0.461	0.750	0.582	0.750	*0.028
CT value variance	0.657 (0.569-0.746)	0.682	0.559	0.783	0.526	0.684	0.679	0.684	*0.030
Model	0.821 (0.753-0.890)	0.775	0.794	0.759	0.595	0.818	0.730	0.818	
Validation cohort									
Density	0.754 (0.641- 0.867)	0.750	0.786	0.722	0.534	0.812	0.688	0.812	*0.049
2D short diameter	0.693 (0.561- 0.825)	0.625	0.571	0.667	0.461	0.750	0.523	0.750	*0.045
CT value variance	0.685 (0.543-0.826)	0.688	0.607	0.750	0.526	0.711	0.654	0.711	*0.048
Model	0.811 (0.693-0.928)	0.781	0.786	0.778	0.595	0.824	0.733	0.824	

AUC: area under curve; CI: confdence interval; Acc: accuracy; Spe: specificity; Sen: sensitivity; PPV: positive predictive value; NPV: negative predictive value.

*:p value was less than 0.05.

## Discussion

AI is increasingly integrated into medical imaging, enhancing the efficiency of lung nodule diagnosis by assisting radiologists in distinguishing benign from malignant nodules. However, the qualitative diagnosis of BA is frequently overlooked in AI-assisted diagnostics, likely due to its low prevalence, despite recent reports of a rising incidence. In our study, we identified density, 2D short diameter, and CT value variance as independent predictors for distinguishing BA from LAC. The predictive nomogram showed good performance in the training and external validation datasets with AUCs of 0.821 and 0.811.

BA is a rare lung tumor, with no reported cases of recurrence or metastasis. Its detection has increased as understanding of its pathology has advanced [17].Located in the peripheral regions of the lungs, BA features a double-layered cellular structure, consisting of a basal cell layer and a luminal cell layer, and grows in a papillary pattern and/or along the alveolar walls in a flat configuration. Tumor of BA appears grayish-white or grayish-brown, soft in texture, and may contain mucus upon sectioning.[6,17,18]. The diagnosis of BA relies on identifying a continuous layer of basal cells, confirmed by IHC using basal cell markers such as p40, p63, and CK5/6 [19,20], and difficult to identify in intraoperative frozen sections. An accurate preoperative diagnosis of BA through imaging can assist in formulating an optimal treatment plan, potentially prioritizing less invasive approaches, such as sublobar resection instead of lobectomy surgery.

In this study, density was identified as an independent risk factor for differentiating BA from LAC on CT images. The results indicated that pure ground-glass and part-solid densities were more likely to be LAC, whereas solid density was more associated with BA. Grossly, most BAs appear as well-demarcated yellowish-white to gray, firm nodules [21]. The alveolar lumens surrounding BA tumors are often filled with mucin, forming mucus pools that manifest as solid densities on CT images. Additionally, due to recurrent internal proliferation and chronic growth patterns [22], BA nodules tend to present as solid densities on CT imaging. In contrast, LAC commonly demonstrates a lepidic growth pattern, in which tumor cells spread along the alveolar walls and progressively invade adjacent parenchyma, vasculature, or pleural structures [23]. This invasive behavior accounts for the typical appearance of LAC as pure ground-glass or part-solid nodules on CT. Recent studies have also shown that CT values are positively correlated with the degree of malignancy in LAC [24,25].

This study found that CT value variance was higher in BA group compared to the LAC group. CT value variance, defined as the squared difference between each sample and the overall mean, indicates data dispersion—larger variance means greater dispersion. Previous studies have shown that the degree of invasiveness in LAC was positively correlated with CT value variance, as the malignancy of the tumor was associated with the complex internal structural components.[14,26–28]. In BA nodules, the increased variance may result from stromal lymphoplasmacytic infiltration, leading to inflammation, hemorrhage, and mucin pooling [29], which collectively contribute to voxel heterogeneity. In contrast, LAC nodules are more cellular and structurally uniform [18], resulting in lower variance. To date, no direct comparison of CT variance between BA and LAC has been reported. Currently, no studies have compared the CT value variance between BA and LAC.

Several studies have suggested that almost all nodules of BA were smaller than LAC [6,30]. Consistent with these findings, our study also showed that the 2D long diameter, 2D short diameter, 2D average diameter, and 2D maximum surface area of BAs were smaller than those of LACs. Among these measurements, the 2D short diameter was identified as an independent predictive factor. BA nodules grown slowly, originating from alveolar cells and being confined by surrounding fibrous septa and bronchovascular bundles. In contrast, the rapid growth of LAC nodules correlated with an increase in diameter [3,24]. Interestingly, our study found that the diameter of BA nodules were smaller than LAC nodules, both on 2D and 3D parameters. It suggested that smaller nodules may indicate the presence of BA. In the training cohort, sphericity was significantly different between the two groups, with the BA group showing a value closer to 1 compared to the LAC group.

In this study, we have demonstrated that BA exhibited lower entropy than LAC nodules. Entropy was a measure of the complexity of image texture and reflect the complex components within the nodules. Previous researches [24,26] have indicated that entropy is a crucial indicator for classifying adenocarcinoma in situ (AIS), minimally invasive adenocarcinoma (MIA) and invasive adenocarcinoma (IAC), with entropy values progressively increasing from AIS to MIA to IAC. The energy value which reflects the magnitude of voxel values within an nodule, has been shown in previous research [24] to be less reliable for predicting the pathological types of lung nodules. Consistent with our findings, the energy of the BA group was lower than LAC in the training set but showed the opposite trend in the validation cohort, suggesting that further investigation with a larger sample size may be warranted. Then, the predictive model was established and improved the accuracy to distinguish between BA and LAC.

This study still has several limitations. Firstly, although the number of BA cases in this study was larger than in previous studies, it remains insufficient for classifying nodules based on different density types. In the future research, we will focus on expanding the sample size to enable a more comprehensive classification. Secondly, the study did not further compare different levels of invasiveness in LAC. Finally, this study focused only on first-order radiomic features, as most currently available AI-assisted diagnostic platforms in clinical practice are based on these features. However, higher-order radiomic features have the potential to better capture tumor heterogeneity. We plan to incorporate these advanced features, along with explainability methods, in future research with a larger cohort to further explore their diagnostic value.

## Conclusion

The predictive model based on AI derived features (density, 2D short diameter and CT value variance), as a non-invasive preoperative examination method, could improve the accuracy in identifying BA and LAC. The nomogram has the potential to become a useful tool for surgeons in formulating accurate surgical plans, thereby improving diagnostic efficiency and patient outcomes in clinical practice.

## Supporting information

S1 File The comparison table of the key distinguishing features between BA and LAC. (ZIP)
