# Supplementary material for: Artificial intelligence-based CT histogram parameters differentiating bronchiolar adenoma and lung adenocarcinomas: A two-center study
Source: PLoS One. 2025 Sep 8;20(9):e0331336. doi: 10.1371/journal.pone.0331336 (PMC12416670; doi:10.1371/journal.pone.0331336)
Supplement: S1 File — (ZIP) [file pone.0331336.s001.zip › Supplementary Information/Supplementary Material.docx]

| Histogram features | BA | LAC | Clinical relevance |
| --- | --- | --- | --- |
| Density | Predominantly Solid | Predominantly ground glass or subsolid | BA typically appears as solid nodules on CT due to mucin accumulation and chronic proliferative growth, whereas LAC more often presents as ground-glass or part-solid nodules, reflecting its lepidic and invasive growth pattern. |
| 2D short diameter | Smaller | Larger | BA nodules grown slowly, originating from alveolar cells and being confined by surrounding fibrous septa and bronchovascular bundles. In contrast, the rapid growth of LAC nodules correlated with an increase in diameter. |
| CT value variance | higher | lower | The squared difference between each sample and the overall mean, indicates data dispersion—larger variance means greater dispersion  The higher variance in BA nodules is likely due to stromal inflammation, hemorrhage, and mucin pooling, whereas the greater cellular uniformity of LAC leads to lower variance. |
| Sphericity | ≈1 | slightly lower | Range from 0 to 1, reflect nodule morphology, with lower values indicating more irregular and potentially more invasive lesions. |
| Compactness | ≈1 | slightly lower |  |
| Kurtosis | lower | higher | Uniformity of nodule density by characterizing the asymmetry of CT values |
| Energy | lower | higher | The magnitude of voxel values in the image. The higher the energy value, the greater the malignancy of the pulmonary nodule. |
| Entropy | lower | higher | Entropy was a measure of the complexity of image texture and reflect the complex components within the nodules. The higher the energy value, the greater the malignancy of the pulmonary nodule. |

Supplementary Table 1. The comparison table of the key distinguishing features between BA and LAC
